# Retraction Note: Surface passivation of CdSe quantum dots in all inorganic amorphous solid by forming Cd_1−x_Zn_x_Se shell

**DOI:** 10.1038/s41598-024-69576-0

**Published:** 2024-08-19

**Authors:** Mengling Xia, Chao Liu, Zhiyong Zhao, Jing Wang, Changgui Lin, Yinsheng Xu, Jong Heo, Shixun Dai, Jianjun Han, Xiujian Zhao

**Affiliations:** 1 State Key Laboratory of Silicate Materials for Architectures, Wuhan University of Technology, 122 Luoshi Road, Hongshan, Wuhan, 430070 People’s Republic of China; 2 Key Laboratory of Photoelectric Materials and Devices of Zhejiang Province, Ningbo, 315211 People’s Republic of China; 3 Photonic Glasses Laboratory, Department of Materials Science and Engineering, Pohang University of Science and Technology, Pohang, Gyeongbuk 790-784 Korea

Retraction of: *Scientific Reports* 10.1038/srep42359, published online 07 February 2017

The Editors have retracted this Article.

After publication of this Article concerns were brought to the attention of the Editors that the exchange reaction described in Equation (1) is not valid, since the preparation process involves melting the mixture at temperatures at which it becomes a homogenous glass in which ZnSe and CdO molecular species are no longer present. Post-publication expert peer review corroborated these concerns and confirmed that the data shown in the article are not sufficient to support the main conclusion about formation of CdSe/Cd_1−x_Zn_x_Se core/graded shell quantum dots. The Editors therefore no longer have confidence in the conclusions of this study.

All Authors agree with this retraction and its wording.

